# One-Pot, Mix-and-Read Peptide-MHC Tetramers

**DOI:** 10.1371/journal.pone.0001678

**Published:** 2008-02-27

**Authors:** Christian Leisner, Nina Loeth, Kasper Lamberth, Sune Justesen, Christina Sylvester-Hvid, Esben G. Schmidt, Mogens Claesson, Soren Buus, Anette Stryhn

**Affiliations:** Laboratories of Experimental Immunology, University of Copenhagen, Copenhagen, Denmark; Centre de Recherche Public-Santé, Luxembourg

## Abstract

**Background:**

Cytotoxic T Lymphocytes (CTL) recognize complexes of peptide ligands and Major Histocompatibility Complex (MHC) class I molecules presented at the surface of Antigen Presenting Cells (APC). Detection and isolation of CTL's are of importance for research on CTL immunity, and development of vaccines and adoptive immune therapy. Peptide-MHC tetramers have become important reagents for detection and enumeration of specific CTL's. Conventional peptide-MHC-tetramer production involves recombinant MHC production, in vitro refolding, biotinylation and tetramerization; each step followed by various biochemical steps such as chromatographic purification, concentration etc. Such cumbersome production protocols have limited dissemination and restricted availability of peptide-MHC tetramers effectively precluding large-scale screening strategies involving many different peptide-MHC tetramers.

**Methodology/Principal Findings:**

We have developed an approach whereby any given tetramer specificity can be produced within 2 days with very limited effort and hands-on time. The strategy is based on the isolation of correctly oxidized, in vivo biotinylated recombinant MHC I heavy chain (HC). Such biotinylated MHC I HC molecules can be refolded in vitro, tetramerized with streptavidin, and used for specific T cell staining-all in a one-pot reaction without any intervening purification steps.

**Conclusions/Significance:**

We have developed an efficient “one-pot, mix-and-read” strategy for peptide-MHC tetramer generation, and demonstrated specific T cell straining comparable to a commercially available MHC-tetramer. Here, seven peptide-MHC tetramers representing four different human MHC (HLA) class I proteins have been generated. The technique should be readily extendable to any binding peptide and pre-biotinylated MHC (at this time we have over 40 different pre-biotinylated HLA proteins). It is simple, robust, and versatile technique with a very broad application potential as it can be adapted both to small- and large-scale production of one or many different peptide-MHC tetramers for T cell isolation, or epitope screening.

## Introduction

Antigen-specific, MHC-restricted T cells (hereafter referred to as “specific T cells”) play many important roles in the generation of immune responses. It is therefore important to be able to identify, enumerate and characterize specific T cells, e.g. during infections, vaccinations or immunotherapies. In the past, assays of proliferation, cytotoxicity, and/or bulk cytokine production have been used as functional evidence of the presence of specific T cells. Combined with laborious limiting dilution experiments, these assays have even provided indirect measurements of the frequency of specific T cells. More recent and less cumbersome assays have detected and counted specific T cell responses at the single cell level using either ELISPOT assays of cytokine release [1]–[4], or flow cytometry-based assays for intracellular cytokine production [5]. These assays, however, are still based upon functional assays and the frequency of responding T cells may not necessarily be the same as the frequency of T cells expressing the appropriate T cell receptor [6]. In a 1996 landmark achievement, Altman and co-workers [7] generated fluorescence labeled, peptide-MHC multimers of defined specificity (generally known in the field of T cell immunology as “MHC tetramers”) and demonstrated their ability to stain specific T cells. They surmised that peptide-MHC multimers would engage several of the T cell receptors expressed on a specific T cell surface and stabilize an otherwise very short lived interaction, thus allowing direct and specific staining of the T cell. Whereas functional assays involves some kind of re-stimulation, MHC tetramers can be used to identify specific T cells without further in vitro manipulation, and they allow for a simultaneous evaluation of the differentiation state through co-staining for various cell surface differentiation markers. In addition, MHC tetramers can be used to purify [8], manipulate, and stimulate [9], [10] specific T cells.

Thus, MHC tetramers provide a simple, fast and efficient approach for monitoring and handling specific T cells. Unfortunately, the production of MHC tetramers is technically demanding. The Altman tetramer production can be sub-divided into three major steps all of which requires biochemical expertise: peptide-MHC monomer production, biotinylation of peptide-MHC monomers, and finally addition of fluorescent labeled streptavidin to effect tetramerization. After each of these steps, the product has to be processed biochemically (e.g. purified by column chromatography, concentrated etc.) before being subjected to the next step. This need for a biochemical set-up and proficiency limits the dissemination of the technology and effectively prevents the application of MHC tetramers to large-scale analysis and use. Indeed, the NIH has created a core facility to generate MHC tetramers; however, the use of the NIH MHC tetramer facility requires approval and may be limited.

We have previously generated pre-oxidized recombinant MHC class I molecules of a quality that allows virtually complete folding and peptide-MHC complex formation[11]. Here, we have generated fully pre-biotinylated versions of the MHC class I molecules and demonstrated that these can be used as convenient reagents in the preparation of biotinylated peptide-MHC monomers. Adding streptavidin to such MHC complexes efficiently leads to MHC tetramer formation. Given the efficiency of our folding, biotinylation and tetramerization processes, we reasoned that it might be possible to omit most, if not all, of the preparative biochemical work involved in tetramer production and create a “one-pot, mix-and-read” protocol. In this scenario, recombinant, pre-biotinylated MHC molecules would be successively reacted with peptide, streptavidin and eventually T cells without any intervening purification or concentration processes. Indeed, we have developed several examples of such “one-pot, mix-and-read” tetramers and demonstrated specific T cell staining of a quality fully comparable with a commercially available counterpart. Pre-oxidized and pre-biotinylated MHC class I heavy chain molecules could be produced in large quantities for wide distribution to laboratories, where they could be readily used to generate tetramers with peptides of choice. Thus, this tetramer technology could allow virtually any end-user to generate his or hers own MHC tetramers at will, and it should allow large-scale MHC tetramer screening approaches.

## Results and Discussion

### Production of recombinant biotinylated MHC-I heavy chains

**Figure 1:**
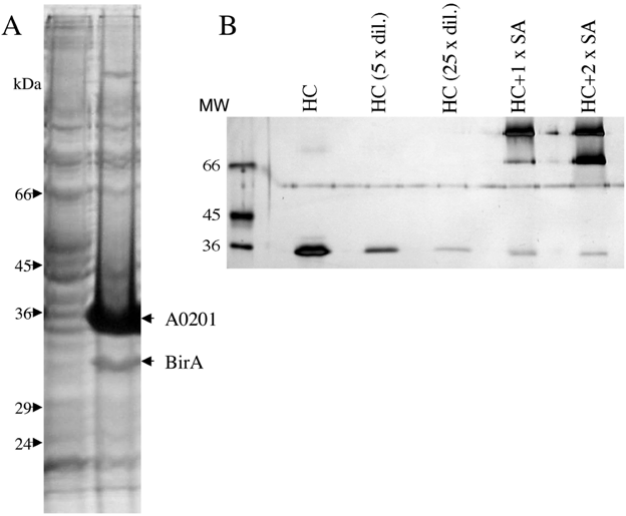
Production of MHC-I heavy chains. Clones of Escherichia coli were selected for high expression of HLA-A*0201-HAT-BSP HC and BirA enzyme and the HLA-A*0201-HAT-BSP product was tested for biotinylation efficiency. In A) Samples of the clone were withdrawn before and after IPTG induction and analyzed on reducing SDS-PAGE. The bacterial cell pellet was resuspended in 50 µL MgCl2/SDS lysis buffer to solubilize heavy-chain inclusion bodies. After centrifugation at 20,000 g for 2 min, 15 µL of the supernatant was loaded directly on the gel. Lane 1, protein marker; lane 2, before induction; and lane 3 after induction with IPTG. Positions of HC monomer and BirA are shown with arrows. In B) HLA-A*0201-HAT-BSP was tested for biotinylation efficiency by a streptavidin gel-shift assay. Purified in vivo biotinylated HLA-A*0201-HAT-BSP incubated for 20 min with either no SA, equal amount of SA, or SA in 2-fold excess. The samples were diluted in 2×Laemmli buffer and electrophoresed on a 12% polyacrylamide gel. For the purpose of quantitative densitometric analysis the HC sample without SA was applied to the SDS-PAGE in 3 different amounts (undiluted, 5-fold, and 25-fold diluted). The percentage of biotinylated HC was determined from the ratio of density of the monomer HC band in the sample with excess SA compared to the band in the diluted sample without SA.

To assure one single specific biotinylation of each recombinant MHC molecule, a biotinylation substrate peptide (BSP) sequence specific for the E.*coli* biotin protein ligase, BirA, enzyme was attached to the C-terminal end of the recombinant MHC-I HC. This enables specific biotinylation of MHC-I HC by BirA. For MHC tetramer production, biotinylation of the BSP site has mostly been based on biochemical in vitro biotinylation of the folded MHC-I complex. This needs to be followed by chromatographic purification steps, which are time consuming and potentially associated with significant loss of material. Induction of efficient in vivo biotinylation of the HC would therefore save both time and material. The endogenous BirA expression in E. coli is insufficient for an efficient biotinylation of over-expressed proteins. However, co-expression of the BirA enzyme either in the E. coli or insect cell expression systems has been shown to increase the biotinylation efficiency[12], [13]. Therefore, the BL21 (DE3) E. coli strain was co-transformed with a pET28a+ vector encoding the MHC-I HC-BSP and a pASYC vector encoding the BirA enzyme. Production of HC and BirA were induced by addition of IPTG for the last 3 h of culture (Figure 1A). Biotin was added to the bacterial culture immediately before induction of expression of HC and BirA. The biotinylation efficiency of the HC was evaluated in a SDS-PAGE gel-shift assay. Samples of HC were incubated with increasing amount of Streptavidin (SA), and subsequently separated by SDS-PAGE. Biotinylated HC will bind to SA and thereby significantly increase the molecular weight of the complex. This resulted in disappearance of the HC-band and appearance of bands of higher molecular weights (Figure 1B). Using densitometry, the residual HC-band representing the non-biotinylated HC were quantified and compared to the total amount of HC (lane without SA added). We have produced and analyzed 27 HLA-A alleles and 14 HLA-B alleles this way, and in all cases obtained biotinylation efficiencies in the range of 85–100% (Table 1). More than ¾ of these preparations were more than 95% biotinylated the rest were between 82 and 94% biotinylated. Thus, the in vivo biotinylation procedure was highly efficient.

**Table 1:** HLA preparations

HLA HC	% biotinylation	% folding
A*0101-BSPHAT	97	96
A*0201-HATBSP	100	95
A*0203-HATBSP	96	ND
A*0204-HATBSP	98	ND
A*0205-HATBSP	98	ND
A*0210-HATBSP	92	ND
A*0211-HATBSP	97	ND
A*0212-HATBSP	96	ND
A*0216-HATBSP	88	ND
A*0219-HATBSP	89	ND
A*1101-BSPHAT	100	ND
A*2301-HATBSP	98	ND
A*2403-HATBSP	98	ND
A*2601-HATBSP	89	ND
A*2602-HATBSP	95	ND
A*2603-HATBSP	97	ND
A*2703-BSPHAT	98	ND
A*2902-HATBSP	97	ND
A*3002-HATBSP	94	ND
A*3303-HATBSP	98	ND
A*4301-HATBSP	100	ND
A*6601-HATBSP	96	ND
A*6802-HATBSP	96	ND
A*7401-HATBSP	100	ND
A*8001-HATBSP	100	ND
B*0702-BSPHAT	100	100
B*0801-BSPHAT	97	ND
MHC HC	% biotinylation	% folding
B*1501-BSPHAT	97	ND
B*1509-HATBSP	94	ND
B*1513-BSPHAT	95	ND
B*1517-HATBSP	100	ND
B*1801-BSPHAT	89	ND
B*2705-BSPHAT	100	ND
B*3901-BSPHAT	94	ND
B*4001-BSPHAT	97	ND
B*4402-HATBSP	97	100
B*5101-HATBSP	100	ND
B*5201-HATBSP	100	ND
B*5301-HATBSP	100	ND
B*5401-HATBSP	98	ND
B*5801-BSPHAT	82	ND

HLA HC denotes the identity of the HLA heavy chain and the order of the attached biotinylation (BSP) and affinity (HAT) tag. The degree of biotinylation and the foldability is determined as stated in Materials and Methods

### Biological activity of biotinylated MHC-I

**Figure 2:**
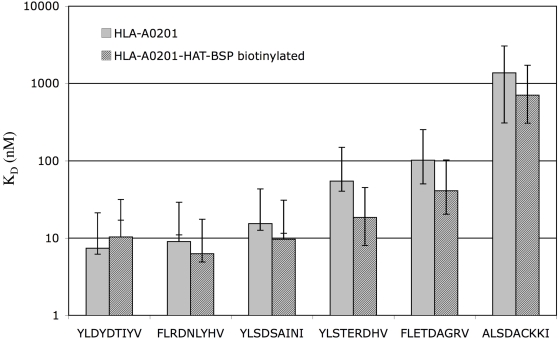
Peptide binding affinity of biotinylated HLA-A*0201 versus non-biotinylated. The peptide binding affinities of the biotinylated HLA-A*0201-HAT-BSP was compared to the standard truncated HLA-A*0201 molecule. The HLA-A*0201 peptide binding affinities of a panel of pox peptides ranging from god binders (0–10 nM) over intermediary (10–100 nM) to non-binders (>1 µM) were determined in a quantitative ELISA [14]. The HC were folded with various concentrations of peptide and excess of hβ_2_m. The K_D_ values for each peptide MHC combination were calculated and the results presented as means of two independent experiments with the corresponding confidence intervals.

To ensure that the in vivo biotinylation did not affect the specificity and subsequent folding of the MHC class I complex, we compared the peptide binding affinities of HLA-HLA-A*0201-HAT-BSP with and without biotinylation to the unmodified recombinant HLA-A*0201. A panel of small pox peptides representing good binders of K_D_<10 nM (YLDYDTIYV, FLRDNLYHV, and YLSDSAINI), intermediate binders of K_D_ 10-100 nM (YLSTERDHV and FLETDAGRV), and non binding peptides of K_D_>1 µM (ALSDACKKI), were analyzed for binding affinity to each of HLA-A*0201 HC constructs. The HLA-A*0201 constructs were folded in the presence of ß_2_m and a titration of the peptides and the K_D_ of each combination was determined in a quantitative ELISA assay [14] (Figure 2). Each peptide, strong as well as poor binders, gave essentially the same K_D_ value for all three HLA-A*0201 constructs. Thus, neither the addition of the BSP site nor the biotinylation prior to folding affected the binding specificity of the HLA-A*0201 molecule.

### Refolding efficiency

In addition to a high biotinylation frequency, our approach also depends upon a high refolding efficiency. To this end we have exploited two previous observations; the first demonstrating that denatured class I MHC heavy chains with pre-oxidized disulfide bonds efficiently refold (when provided with β_2_m and an appropriate peptide)[15], and the second demonstrating that the efficiency of this process can be further improved by purification of denatured heavy chain molecules with correct disulfide conformations[11]. Thus, we have previously shown that subspecies of correctly oxidized recombinant denatured MHC-I HC can be purified from the inclusion bodies, and that these upon dilution folds into functional complex with a >90% efficiency[11]. In this paper, we have used 4 different MHC-I HC preparations for tetramer production: HLA-A*0101, HLA-A*0201, HLA-B*0702, and HLA-B*4402. These HC's were in vivo biotinylated, purified, and subsequently folded in the presence of a relevant peptide, pp65_363–373_, pp65_495–503_, pp65_417–426_, and EBNA3b_657–666_ respectively. These complexes were titrated and measured in a quantitative ELISA. The folding efficiency was determined by comparison to a standard of known complex concentration. As seen in Table 1, we obtain 95–100% correctly folded complex and these HC were 97–100% biotinylated. Note, that although we don't know where and how these disulfide bonds are generated, we have at this point successfully generated tetramers from HLA preparations representing 14 different HLA alleles, and in no case have we failed. We conclude that these molecules in principle are ready to be tetramerized with SA without further purification.

### Tetramerization of MHC and streptavidin

**Figure 3:**
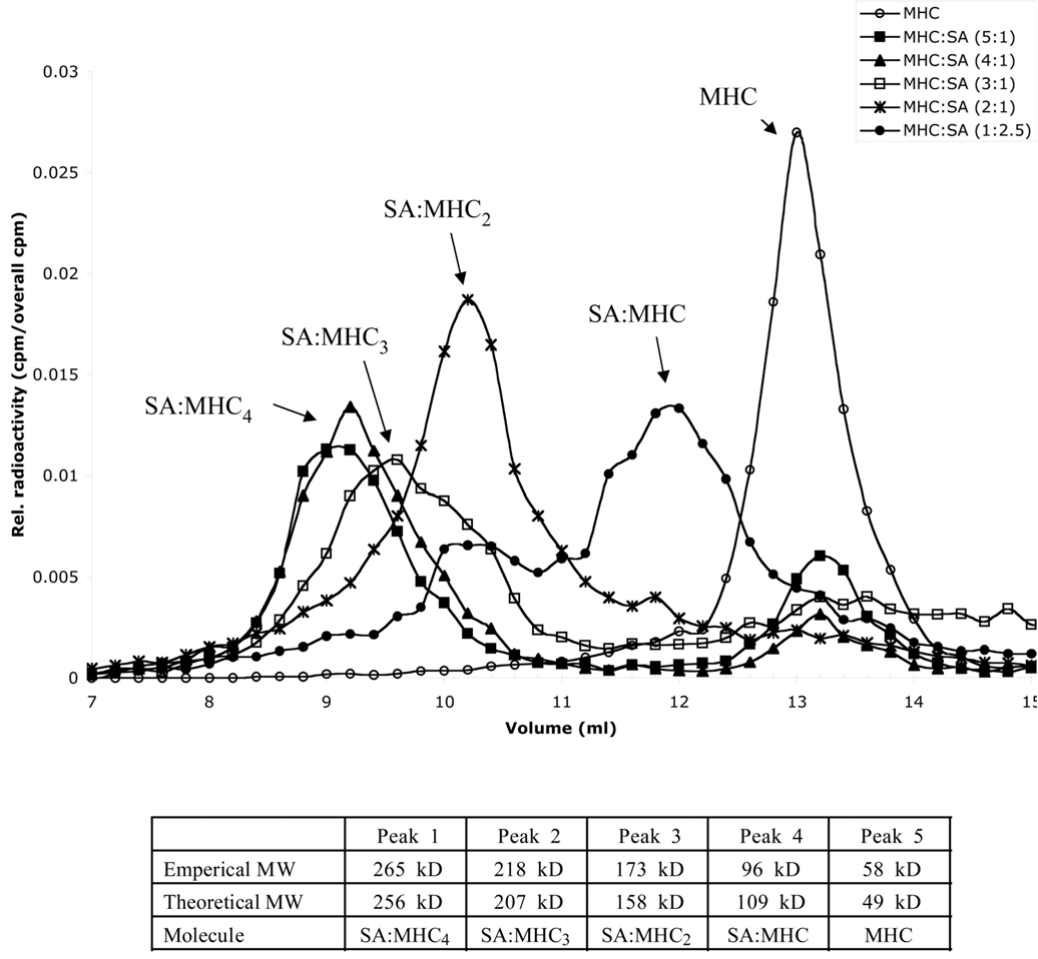
Binding of SA and biotinylated HLA-A*0201-peptide complexes. The folding reaction of biotinylated HLA-A*0201 was spiked with radioactively labeled peptide. These complexes were reacted with SA at various different molar ratios in order to produce MHC-I tetramers, trimers, dimers, and monomers. The various SA-MHC-I complexes were run on a calibrated SEC column. The eluate fraction of the SA-MHC-I complexes was determined through radioactivity measurements. (A) Size exclusion chromatogram of 6 different stoichiometric mixtures of SA to HLA-A*0201-peptide complex. In (B) is listed the calculated MW's corresponding of the individual peaks of the chromatogram.

In order to follow the stoichiometric distribution of SA-MHC-I complexes during the formation of peptide-MHC tetramers, the folding reaction of biotinylated HLA-A*0201-HAT-BSP was spiked with radioactively labeled peptide. MHC-tetramers were the generated at different molar ratios of MHC-I and SA; and the formations of various SA-MHC-I multimers were followed by size-exclusion chromatography (SEC) through detection of radioactivity (Figure 3). SA binds MHC-I at a 1 to 4 molar ratio. Gradually increasing the SA to MHC-I ratio from MHC-I in excess to SA in excess, saturated and unsaturated SA molecules could be generated at different stoichiometric ratios; SA:MHC_4_, SA:MHC_3_, SA:MHC_2_, SA:MHC, and eventually MHC monomers. Quantification of molecular weights according to standard marker proteins showed agreement between theoretical and empirical molecular sizes (Figure 3). All four SA-MHC-I multimers were generated dependent upon the ratio between SA and MHC-I. At SA to MHC-I ratio of 1:4, the tetramer form was almost quantitatively produced, generating none of the other multimer species and only leaving a small fraction of non-reacted monomers. This presumably represents a small fraction of non-biotinylated HC.

### Staining specific T cells using “one pot, mix and read” tetramers

The efficiency of the biotinylation and folding processes suggested to us that it should be possible to generate MHC tetramers in a “one-pot” reaction omitting most, if not all, of the preparative biochemical work normally involved in tetramer production. To analyze whether our “one-pot” tetramers were able to stain specific T cells in a “mix-and-read” mode, we initially selected the HLA-A*0201 restricted dominant CMV T cell epitopes, CMV pp65_495–503_ peptide as model system. CMV pp65_495–503_ specific T cells from healthy donors were generated and HLA-A*0201/pp65_495–503_-SA-phycoerythrin (PE) tetramers were produced as described above. The tetramers were not concentrated nor further purified, but used directly to stain the specific T cells.

**Figure 4:**
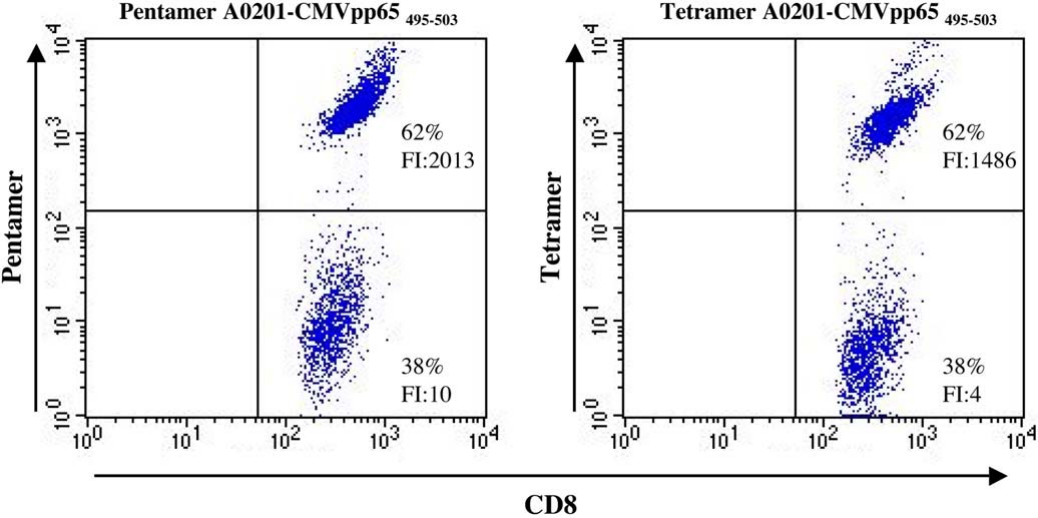
Tetramer staining comparison to pentamer. PBMC from a healthy donor were stimulated for 8 day with the pp65_495–503_ peptide and stained with APC labeled anti-CD8 and PE-labeled HLA-A*0201-pp65_495–503_ tetramer (right panel) or with PE-labeled HLA-A*0201-pp65_495–503_ pentamer (left panel). The dot plot shows the gated CD8 T cells. Numbers in the upper right quadrant of each plot are the percentage of epitope specific CD8 T cells. The fluorescence intensity (FI) of the staining of the positive and negative population is given in the upper and lower right quadrant, respectively.

The specificity and sensitivity of the tetramer staining were compared to that of the commercially available HLA-A*0201/pp65_495–503_ pentamers (®ProImmune, www.proimmune.com) (Figure 4). The tetramers stain about 60% of the CD8 T cells with a baseline separation from the negative population. The same frequency of specific T cells was detected using the commercial pentamers. The staining intensity of the tetramers were slightly lower than that of the pentamers (FI 1480 vs. 2042), however, the background staining of the negative population was also lower (FI 4 vs. 10) resulting in a higher signal to noise ratio for the in-house generated tetramers (370 vs. 202). Thus the “one-pot, mix-and-read” strategy for the HLA-A*0201/pp65_495–503_ tetramer production appeared to work as well as the MHC pentamer of the same specificity.

**Figure 5:**
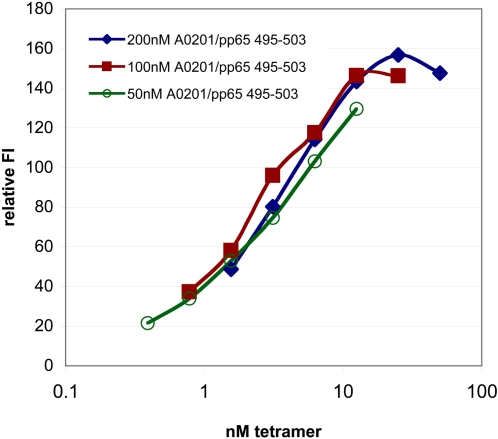
Titration of complex concentration for tetramer production. Three different concentrations of HLA-A*0201-pp65_495–503_ complexes were folded (50, 100, and 200 nM) and tetramers were produced from these. PBMC from a CMV pp65_495–503_ responsive donor (3.5% specific CD8 T cells) were stained with a titration of each of the three tetramer productions. The x-axis is the actual calculated concentration of tetramers in the cell staining reaction. The results are depicted as the relative fluorescence intensity (FI signal of the positive population/FI signal of the negative population).

The MHC-I monomers were produced at a rather low concentration compared to conventional tetramer production. These monomers were mixed with streptavidin directly, without any concentration, to generate the tetramers. The stability, yield, and robustness of this production strategy were of concern. We found that the tetramers could be stored for more that 9 months at 4°C with no change in activity (data not shown). Another concern was whether there was a lower concentration limit of monomers needed for production of tetramers in order to give an optimal staining of the specific CD8 T cells. To analyze this HLA-A*0201/pp65_495–503_ monomers were folded at 200 nM, 100 nM and 50 nM and each mixed with streptavidin to produce tetramers. Thus, the maximum tetramer concentration of the 200 nM monomers was 50 nM tetramer, of the 100 nM monomers 25 nM tetramer, and of the 50 nM monomers 12.5 nM tetramer. Two fold titrations were made of each of these three productions and used to stain pp65_495–503_ specific T cells. Baseline separation between the positive and negative cell population was seen for all the concentrations used. The relative fluorescence intensity is depicted as a function of the actual tetramer concentration used to stain the cells (Figure 5). The curves for the three-tetramer productions were super imposable showing considerable robustness in set-up and staining reactions. The actual tetramer concentration used in the staining reaction, and not the concentration of monomers used for the tetramer production, was important for the staining intensity. The staining intensity reached a plateau and the T cell labeling was saturated at tetramer concentrations above 12.5 nM. Therefore, saturable T cell staining can be achieved using tetramers produced from concentration of monomers higher than 50 nM.

### Unreacted monomers do not inhibit HLA tetramer staining

**Figure 6:**
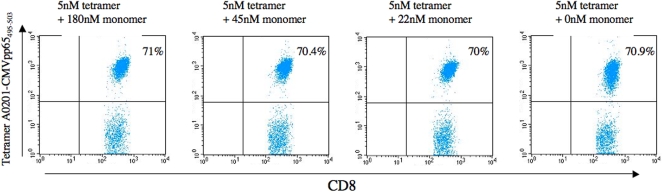
Unreacted monomers do not inhibit tetramer staining. PBMC from a healthy donor was re-stimulated for 8 day with the CMV pp65_495–503_ peptide. They were washed and adjusted to a final concentration of 2.5 million cells/ml containing the indicated concentration of CMV pp65_495–503_/HLA-A*0201 monomer. After 5 min incubation at room temperature, a final concentration of 5 nM CMV pp65_495–503_/HLA-A*0201 tetramer was added. The cells were further stained for CD8 and analyzed by flow cytometry as described in materials and methods.

It is inherent to the “mix-and-read” tetramer strategy that the staining reaction is done with the entire tetramerization reaction mixture including any unreacted monomer such as that of any un-biotinylated HLA molecule. A priori, the tetramerized peptide-HLA complexes would have a much stronger reaction with the appropriate T cell receptor than any unreacted monomers would have. Addressing whether the monomers could inhibit tetramer staining, we added increasing concentrations (up to 180 Nm) of monomers to specific T cells, incubated the cells at room temperature for 5 minutes, and then added a fixed concentration of tetramer (5 nM tetramer corresponding to 20 nM monomer). In no case was any inhibition of tetramer staining observed (Figure 6) suggesting that unreacted monomers do not compromise staining with our one-pot, mix-and-read tetramers. Note, that we have not addressed any physiological effect of peptide-HLA monomers such as the partial activation demonstrated by Malissen and co-workers [16].

### Extending to other HLA-A*0201-peptide epitopes

**Figure 7:**
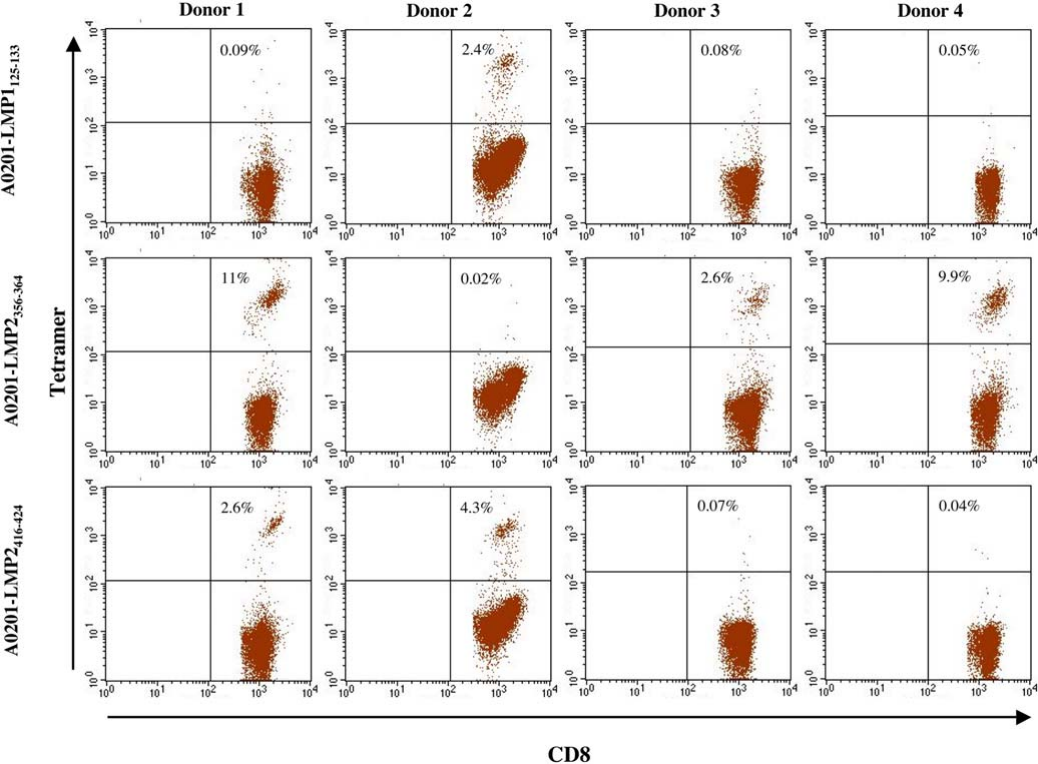
HLA-A*0201-tetramer screening of EBV reactive donors. PBMC from four healthy donors were each re-stimulated for 8 day with three different EBV peptides, LMP1_125–133_, LMP2_356–364_, or LMP2_416–424_, and stained with both HLA-A*0201-LMP1_125–133_ (top panel, HLA-A*0201-LMP2_356–364_ (middle panel) and HLA-A*0201-LMP2_416–424_ tetramers (bottom panel). The dot plot shows gated CD8 T cells and the percentage of tetramer labeled CD8 T cells are given in the upper right quadrant of each plot.

To validate the generality of the “one-pot, mix-and-read” strategy for tetramer production, a different set of tetramers were produced from 3 different HLA-A*0201 EBV peptide complexes: HLA-A*0201/LMP1_125–133_, HLA-A*0201/LMP2_356–364_ and HLA-A*0201/LMP2_416–424_. PBMC's from four different HLA-A2 positive donors were restimulated with the three different peptides for 8 days and then stained with the tetramers (Figure 7). PBMC's from all four donors could be stained by one or more of the three tetramers. In all cases, the specific staining showed baseline separation between positive and negative populations. Three of the donors had LMP2 _356–364_ specific T cells varying from about 3 to 11% of the CD8 T cells being labeled with HLA-A*0201/LMP2 _356–364_. Two of the donors were positive for LMP2 _416–424_ with about 3 and 4% specific CD8 T cells. Only one out of four donors was positive for LMP1_125–133 _with about 3% specific CD8 T cells. The same results were obtained when the frequency of peptide specific T cells producing IFNγ was evaluated by intracellular staining (data not shown). Thus this “one-pot, mix-and-read” strategy can be used to produce several different HLA-A*0201 tetramers and can directly be used for epitope screening analysis.

### Extending to other HLA-alleles

**Figure 8:**
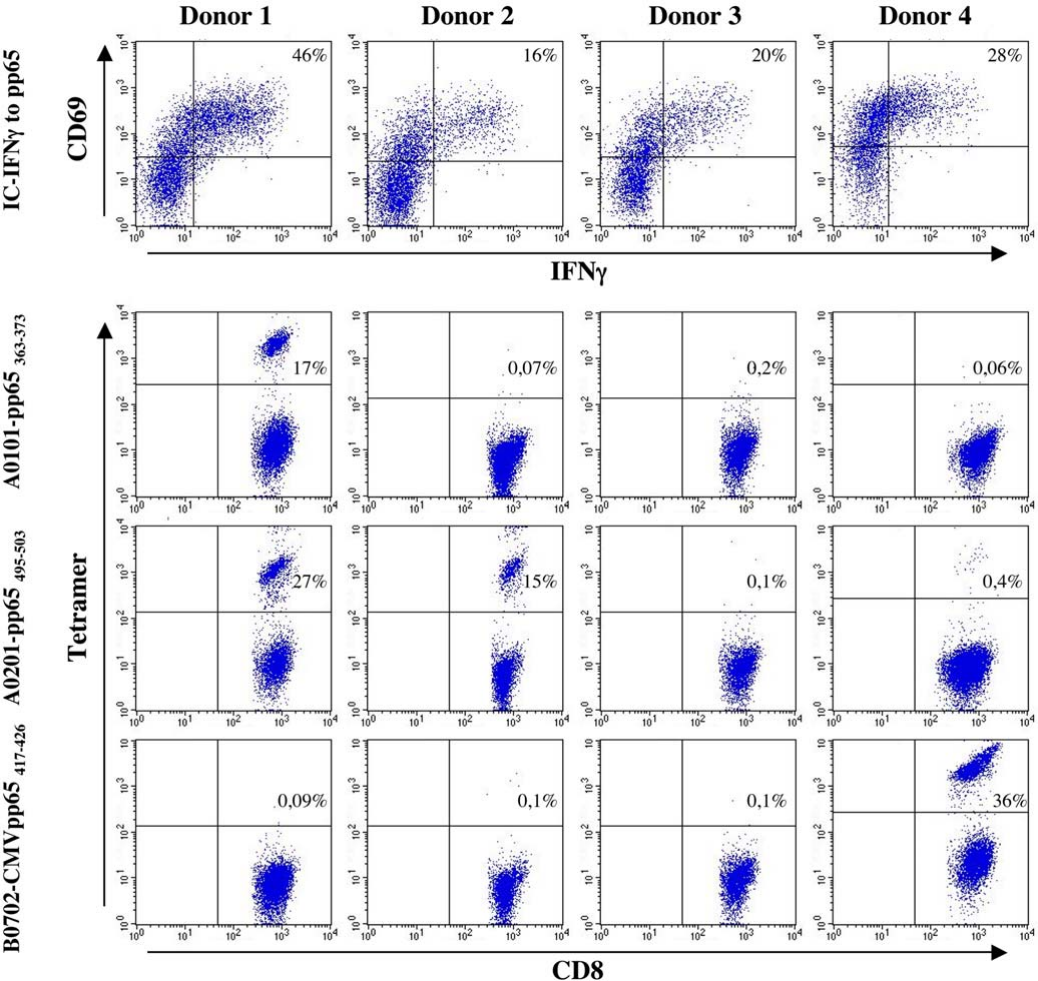
Screening for CMV specific T cells with three different Class I-tetramers. PBMC from 4 healthy CMV pp65 responding donor were stimulated for 8 day with a mixture of 15 amino acid long overlapping peptides spanning the entire pp65 protein. The cells were analyzed pp65 specific responses using the IC-IFNγ assay and stained for CD8, CD69 and intracellular IFNγ (top panel). The donors were screened for HLA-A*0101 restricted pp65_363–373_, the HLA-A*0201 restricted pp65_495–503_, and the HLA-B*0702 restricted pp65_417–426_ specific T cells. Staining the cells with CD8 and each of the three tetramers, HLA-A*0101-pp65_363–373_, HLA-A*0201-pp65_495–503_, and HLA-B*0702-pp65_417–426_. The dot plot shows the gated CD8 T cells. Numbers in the upper right quadrant of each plot are the percentage of epitope specific CD8 T cells.

We wanted to extend this application to other HLA and peptide-HLA combinations. Complexes of two dominant CMV pp65 T cell epitopes, HLA-A*0101/pp65_363–373_ and HLA-B*0702/pp65_417–426_, were folded. As for the HLA-A*0201, the folding efficiency of these complexes were >96% (Table 1). Tetramers of these complexes and the HLA-A*0201/pp65_495–503_ were produced and used to analyze the specificity of a CMV response. PBMC's from four different healthy donors were stimulated for 8 days with a mixture of 134 overlapping 15-mer peptides representing the entire pp65 CMV protein and subsequently analyzed for pp65 specific responses using intracellular IFNγ (IC-IFNγ) staining. All four donors showed a pp65 specific response, having between 16% and 46% pp65 specific CD8 T cells (Figure 8, top panel). Using the HLA-A*0101/pp65_363–373_, HLA-A*0201/pp65_495–503_, and HLA-B*0702/pp65_417–426_ tetramers these donors were analyzed for the specificity distribution against these three dominant pp65 T cell epitopes (Figure 8). All three tetramers showed specific staining and we were clearly able to differentiate the donors as responder and non-responder to the given epitope. Two of the four donors (Donor 1 and 4; Figure 8) stained positive for the HLA-A*0101/pp65_363–373_ tetramer; one having a relative high frequency of positive CD8 T cells and the other a very low frequency (17.6% versus 0.06%). Two donors (Donor 1 and 2; Figure 8) stained positive for the HLA-A*0201/pp65_495–503_, tetramer, both with relative high frequencies (28.5 and 15.6%). Only donor 4 stained positive for HLA-B*0702/pp65_417–426_ with 36% of the CD8 T cells being specific for this epitope. Except for one donor, the frequencies of tetramer positive CD8 T cells for each donor fully covered the entire pp65 response measured by intracellular staining for that donor. Donor 3 was not stained by any of the three tetramers. Therefore, the pp65 specific CD8 T cells response, must be represented by pp65 epitopes different from the three used for tetramers.

### Correlating staining with functionality

**Figure 9:**
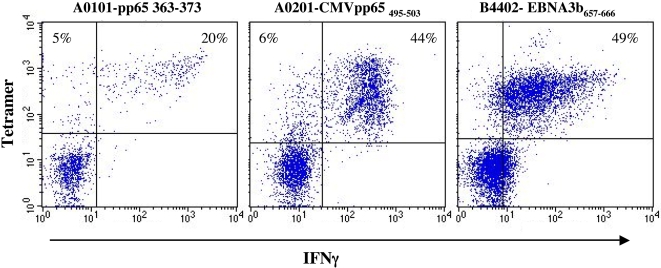
Double staining with tetramer and IC-IFNγ. Τhree donors responding to either HLA-A*0101 restricted pp65_363–373_, HLA-A*0201 restricted pp65_495–503_, or HLA-B*4401 restricted EBV EBNA3b_657–666_, were restimulated with the respective peptides for 3 h in the presence of Brefeldin A. The cells were stained with the relevant tetramers, HLA-A*0101-pp65_363–373_, HLA-A*0201-pp65_495–503_, or HLA-B*4401-EBNA3b_657–666_, prior to the staining for CD8 and intracellular IC-IFNγ. The dot plot shows the gated CD8 T cells.

Finally, we correlated tetramer staining with functionality. Though the frequency of tetramer positive cells and IFNγ producing cells correlated nicely we wished to assure that the cells, which stained tetramer positive, were in fact the IFNγ producing cells. Three different tetramers were produced: HLA-A*0101/pp65_363–373_, HLA-A*0201/pp65_495–503_, and B*4401/EBNA3b_657–666_. Three different donors were restimulated for 8 day with the three peptides, CMV pp65_363–373_, CMV pp65_495–503_, and EBV EBNA3b_657–666_, respectively. T cells from each donor were double labeled for tetramer and IC-IFNγ (Figure 9). For all combinations, the specific IFNγ producing CD8 T cells were also stained with the relevant tetramers. For the two donors specific for pp65_363–373_ and pp65_495–503_, a few percentage of the tetramer labeled cells did not produce IFNγ. Since, the majority of the CMV specific CD8 T cells produced both IFNγ and TNFα and only a few percentage produced either one or the other alone, these tetramer+, IFNγ- cells were most likely TNFα producing. Thus, the CD8 T cells stained with these tetramers were in fact the specific CD8 T cells that produce IFNγ in response to the relevant peptide epitope.

### Conclusion

In summary, we have developed a rapid “one-pot, mix-and-read” strategy for tetramer production. Due to highly efficient biotinylation, folding, and tetramerization processes, all the preparative biochemical work normally needed during MHC tetramer generation can be omitted and the production can run as a fast sequential addition of the relevant reagents. Others have also attempted to generate a high-throughput production of MHC class I tetramers[17]. Their strategy is based on peptide exchange of MHC complexes folded with an UV cleavable peptide. These degradable complexes are produced in the conventional way being time consuming and associated with significant loss of material. At this point, this peptide cleavable approach has been applied to one human MHC class I allele (HLA-A*0201) and a mouse allele (D^b^). The broad application of their strategy is limited by the need to define a unique UV-labile epitope for each MHC class I allele. In contrast, our approach is based on the ability to produce fully in vivo biotinylated and readily foldable MHC I HC. Ready-to-use tetramers with the desired specificity and scale can be produced within 2 days in any lab independent of special biochemical skills or equipment. We have demonstrated the application of our “one-pot, mix-and-read” strategy for 7 different MHC I restricted T cells epitopes spanning 4 different HLA class I alleles. We have produced an additional 37 fully biotinylated HLA-class I HC using this strategy (Table 1). This procedure is suitable for large-scale production of a single peptide-MHC tetramer for repeated analysis or purification of specific T cells as well as for a small-scale production of a larger number of different class I-peptide complexes e.g. for a comprehensive T cell screening program.

## Materials and Methods

### Peptides

Peptides were synthesized by standard 9-fluorenylmethyloxycarbonyl (FMOC) chemistry, purified by reversed-phase high-performance liquid chromatography (at least 80%, usually >95% purity) (Shafer-N, Copenhagen, Denmark). Mixes of 134 15 amino acid long peptides spanning the entire CMV pp65 were obtained from PT Peptide Technologies GmbH, Germany.

### Production of human MHC-I heavy chains

Recombinant MHC-I heavy chains (HC's) have been generated from synthetic codon optimized and truncated genes without the transmembrane region. Sequences encoding HAT (a histidine affinity tag) and BSP (a biotinylation tag) were fused to the 3′ end of the truncated HC gene and cloned into a pET28a+ *E. coli* expression vector (Novagen). For expression, the BL21 (DE3) *E. coli* strain was co-transformed with a pET28a+ vector encoding the tagged HC's and a pASYC vector encoding the BirA biotinylation holoenzyme. Clones producing high amounts of HC and BirA upon isopropylthiogalactoside (IPTG) induction were selected. 100 mg/L biotin (Sigma) was included during the 3h IPTG induction.

Large-scale production and purification of proteins were performed as previously described[11]. Where and how the disulfides of the HLA heavy chains are formed is not known. The details of protein handling may be extremely important, including pH, temperature and time. We have not examined these parameters, however, preparations of tetramer generating quality were consistently generated.

### Biotinylation assay

The level of biotinylation of the purified HC was examined by a streptavidin gel-shift SDS-PAGE assay. Purified HC was incubated for 20 min at RT with a functional excess of streptavidin (SA, each streptavidin molecule can bind four biotinylated molecules), and analyzed by 12% polyacrylamide gel (SDS-PAGE, without boiling the samples). SA interacts with biotinylated HC and shifts the approximate 36 kDa monomer band to approximately 100 kDa. For comparison, serial dilutions of HC, which have not been reacted with SA, were analyzed. The intensities of the SA reacted and non-reacted HC bands were determined by densitometric analysis using 1D Gel Analysis software (Kodak Digital Science). The percentage of biotinylated HC could be calculated from the ratio of intensity of the HC band of the sample without SA compared to the sample with excess SA.

### Quantitative measurement of peptide MHC-I complex formation

The peptide binding affinity of biotinylated versus non-biotinylated HLA-A*0201 were compared using our previously described quantitative ELISA assay[14]. Briefly, peptide-MHC-I complexes were folded at 18°C for 48 hours with a constant concentration of hß_2_m and a titration of peptide concentrations. The complex formation was measured with a sandwich ELISA using the conformationally sensitive monoclonal antibody, W6/32, for capturing peptide-MHC monomers and a HRP conjugated polyclonal rabbit anti-human ß_2_m antibody (DAKO) for detection of correctly folded complexes. The ELISA was developed with EnVision™ (DAKO #K4003). MHC-I concentration was measured by spectrophotometric analysis in a Microplate Scanning Spectrophotometer, Discovery HT-R (BIO-TEK Instruments). A peptide-MHC monomer complex of known concentration was used as reference for conversion of absorbance data to concentrations. A sigmoid fit (y = B_max_*x/(K_D_+x) of each peptide titration curve on semi logarithmic coordinate scales provides B_max_ and K_D_ values.

### Size exclusion chromatography of peptide-MHC tetramers

The tetramerization of peptide-MHC-I monomer complexes with SA was visualized by Superdex 200 (Amersham Biosciences) chromatography. Iodinated (^125^I) peptide-MHC-I monomers were generated using radioactively labeled peptides, and subsequently used to spike a preparation of unlabeled peptide-MHC-I monomers. The spiked peptide-MHC-I complexes were then reacted with various stoichiometric ratios of SA. A 100 µl sample of MHC-SA complexes were analyzed on a 23 ml pre-packed and calibrated Superdex 200 SEC column; 200 µl fractions were collected, and analyzed by gamma-spectrometry (Cobra 5010, Packard).

### “One-pot” MHC tetramer production

Peptide-MHC-I monomer complexes were generated by diluting the HC (typical stock concentration 15–40 µM)) 100-fold into a reaction buffer containing 50 mM tris-maleate pH 6.6, 0.1% pluronic F86 NF (BASF, a surfactant compatible with cellular use), a 20-fold molar excess of ß_2_m, and 50-fold molar excess of peptide. The mixture was vortex'ed briefly and incubated at 18°C for 48 hours. Streptavidin-R-Phycoerythrin (SA-PE, Becton Dickinson) was then added (sequentially in 10 small aliquots over 90 min) at a molar ratio of four MHC to one SA. This reaction mixture containing tetramers was stored at 4°C.

### Cell lines and culture condition

Buffy coats were obtained from healthy volunteer Danish donors from The Blood Bank at the national Hospital (Copenhagen, Denmark). Peripheral blood mononuclear cells (PBMC) were isolated by density gradient centrifugation using Ficoll-Paque (GE-healthcare). To stimulate specific CTL, 2 µg/ml peptide were added to the PBMC and incubated for 8–9 days in Xvivo15 (Lonza) supplemented with 5% autologous serum. IL2 at 50 U/ml were added at day 1, 4, and 6. Dendritic cells (DC) were used as APC. PBMC were adhered for 2 h at 37°C in 24-well plates. Adherent cells were cultured for 9 days in Xvivo15 with 5% AB serum, 100 ng/ml GM-CSF, and 100 ng/ml IL4. At day 7 the DC were activated with a mixture of 10 ng/ml TNFα, 5 ng/ml IL1β, 20 ng/ml IL6, and 1 µg/ml PGE2.

### Tetramer staining

PBMC or T cells, harvested day 8, were aliquoted at 2*10^5^/well in a 96-well round bottom microtiter well. The cells were pelleted and resuspended in 25 µl tetramer, incubated for 20 min at RT followed by 30 min incubation with anti CD8-APC (Pharmingen) at 4°C. After two washes the cells were analyzed on a FACScalibur.

### Intracellular staining for IFNγ

DC's were pulsed with or without peptide for 1 h and added to the T cells. The cells were incubated for 3h with Brefeldin A at 37°C. Subsequently, staining for CD8, CD69 and intracellular IFNγ was carried out according to the Fast Immune protocol (Pharmingen). In some cases prior to the Fast Immune staining the cells were stained with tetramers as described above. The cells were analyzed on a FACScalibur.
